# Prognostication after intracerebral hemorrhage: a review

**DOI:** 10.1186/s42466-021-00120-5

**Published:** 2021-05-03

**Authors:** Jens Witsch, Bob Siegerink, Christian H. Nolte, Maximilian Sprügel, Thorsten Steiner, Matthias Endres, Hagen B. Huttner

**Affiliations:** 1 Department of Neurology, Weill Cornell Medicine, 525 East 68th Street, New York, NY 10065 USA; 2 Center for Stroke Research Berlin, Charité Universitätsmedizin, Berlin, Germany; 3 Klinik und Hochschulambulanz für Neurologie, Charité Universitätsmedizin Berlin, Berlin, Germany; 4 Department of Neurology, Universitätsklinikum Erlangen, Erlangen, Germany; 5 Department of Neurology, Klinikum Frankfurt Höchst, Frankfurt a. M., Germany; 6 Department of Neurology, Universität Heidelberg, Heidelberg, Germany; 7 German Center for Neurodegenerative Diseases (DZNE), Partner Site Berlin, Berlin, Germany; 8 German Centre for Cardiovascular Research (DZHK), Partner Site Berlin, Berlin, Germany

**Keywords:** Cerebrovascular disease, Stroke, Intracerebral hemorrhage, Prognosis, Outcome research

## Abstract

**Background:**

Approximately half of patients with spontaneous intracerebral hemorrhage (ICH) die within 1 year. Prognostication in this context is of great importance, to guide goals of care discussions, clinical decision-making, and risk stratification. However, available prognostic scores are hardly used in clinical practice. The purpose of this review article is to identify existing outcome prediction scores for spontaneous intracerebral hemorrhage (ICH) discuss their shortcomings, and to suggest how to create and validate more useful scores.

**Main text:**

Through a literature review this article identifies existing ICH outcome prediction models. Using the Essen-ICH-score as an example, we demonstrate a complete score validation including discrimination, calibration and net benefit calculations. Score performance is illustrated in the Erlangen UKER-ICH-cohort (NCT03183167). We identified 19 prediction scores, half of which used mortality as endpoint, the remainder used disability, typically the dichotomized modified Rankin score assessed at variable time points after the index ICH. Complete score validation by our criteria was only available for the max-ICH score. Our validation of the Essen-ICH-score regarding prediction of unfavorable outcome showed good discrimination (area under the curve 0.87), fair calibration (calibration intercept 1.0, slope 0.84), and an overall net benefit of using the score as a decision tool. We discuss methodological pitfalls of prediction scores, e.g. the withdrawal of care (WOC) bias, physiological predictor variables that are often neglected by authors of clinical scores, and incomplete score validation. Future scores need to integrate new predictor variables, patient-reported outcome measures, and reduce the WOC bias. Validation needs to be standardized and thorough. Lastly, we discuss the integration of current ICH scoring systems in clinical practice with the awareness of their shortcomings.

**Conclusion:**

Presently available prognostic scores for ICH do not fulfill essential quality standards. Novel prognostic scores need to be developed to inform the design of research studies and improve clinical care in patients with ICH.

**Supplementary Information:**

The online version contains supplementary material available at 10.1186/s42466-021-00120-5.

## Background

Among all deaths caused by neurological disease worldwide two thirds are related to stroke [1]. Spontaneous intracerebral hemorrhage (ICH), the most lethal stroke subtype, has shown a remarkable increase in incidence over the past decades to currently approximately 176 cases per 100,000 person-years [2]. Although ICH accounts for only 10–15% of stroke cases it is associated with about 70% of 1-month stroke mortality. Approximately half of patients with ICH die within the first year [3]. Because of the high likelihood of unfavorable outcome after ICH, clinicians are often forced to develop a clinical strategy that is determined by both medical and ethical considerations.

Prognostic models may guide health care providers and families when they are confronted with these complex decisions. However, recent outcome research suggests that current clinical scores, typically designed to estimate the risk of mortality or disability, are neither accurate, nor are they predicting those endpoints that matter to patients and families [4]. The discrepancy between what prognostic scores offer and what is needed in clinical practice has led physicians to increasingly neglect these scores. This trend is fueled by research showing that subjective clinical judgment may be superior to prediction model estimates [5–7].

Here we will review current ICH prognostication tools and point out their shortcomings. The unused potential of new predictor variables and a growing movement towards patient reported outcome measures (PROMs) highlight the need for novel ICH prediction scores. We delineate concepts on how to create and validate new scores, and how to manage and reduce major biases. Lastly, we discuss how currently available scores, despite their shortcomings, can facilitate meaningful prognostication and informed decision-making in clinical practice.

## Main text

### Methods

#### Literature search

We conducted a literature review searching pubmed up to December 31, 2020. Studies were selected according to the CHARMS checklist [8]. We included studies in humans reporting prognostic scores regardless of type or timing of predicted endpoints. The search algorithm and detailed inclusion and exclusion criteria are reported in the online supplement.

#### Complexity of prediction scores

To quantify the ease of use of current scores, we assigned an availability factor to each score component with greater numbers indicating more difficult to obtain data (lower availability). Easily accessible clinical information, e.g., the patient’s age, Glasgow Coma Scale (GCS), serum glucose, or ICH location, was assigned a value of 1. Complex imaging information requiring the use of a software or additional radiological knowledge, e.g., ICH or IVH volume, was assigned a value of 2. Past medical history information was assigned a value of 3. Imaging or physiological scores integrated into a prediction score as one variable, e.g., the APACHE score, were assigned a value of 4. The sum of the weighting factors was then plotted for each prediction score.

**Fig. 1:**
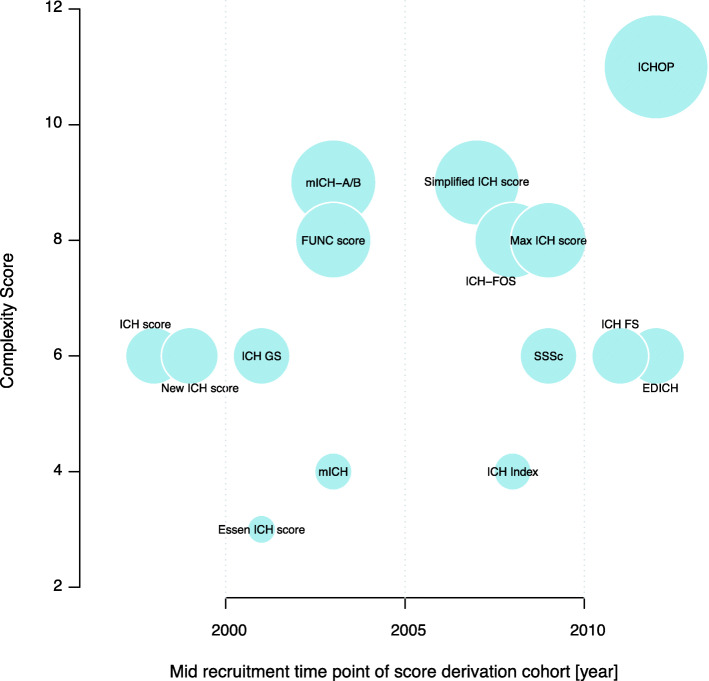
Ease of clinical use of ICH prediction scores. Size of bubbles encodes the degree of complexity of the respective scoring system depending on number and availability of individual score components (see Methods section)

#### Examples score validation

To illustrate the steps of a complete score validation, we calculated and validated the Essen-ICH-score in the UKER ICH-cohort (*n* = 1166). We chose the Essen-score because its original publication was the only score that reported absolute and relative outcome risks [9]. Characteristics of the UKER ICH-cohort have been described previously and are available online at clinicaltrials.gov (NCT03183167) [10]. The Essen-score is designed to predict favorable functional outcomes, as defined by Barthel index > 95. Because the Barthel index was not available in the Erlangen cohort, we assessed the performance of the Essen-score with regards to prediction of favorable functional outcome, defined as modified Rankin score (mRS) 0–3, at 90 days, and survival at 90 days (mRS 0–5).

#### Standard protocol approvals, and patient consents

The UKER ICH-cohort study was approved by the ethics committees and institutional review boards based on the vote from Friedrich-Alexander-University Erlangen-Nuremberg, Germany. Consent was obtained by patients or legal representatives.

## Results

### Literature review

The search returned 4867 pubmed hits, 3825 of which were studies conducted in humans. Title and abstract of these 3825 references were screened, and 3792 papers were excluded after the first screening. The remaining 33 referenced papers were read in full. Among these, 19 studies met inclusion criteria (Table 1). Specific reasons for excluding the other 14 studies are reported in the online data supplement. An inclusion/exclusion flow chart is available in the online Supplement (eFigure 1 in the online supplement).

**Table 1:** Summary of Literature Search: scores to prognosticate outcome after spontaneous intracerebral hemorrhage

Study (First Author, publication year)	Score name	Geographic location of derivation cohort (N of score derivation cohort)	Score components	Outcome measure(s)	Timing of outcome measures (in original score publication)	Score performance measures (in original score publication)
Tuhrim et al., 1988 [11]	No name	USA (82)	GCS ICH volume Pulse pressure	Mortality	30 days	Expected-observed classification
Tuhrim et al., 1991 [12]	No name	USA (191)	GCS ICH volume IVH Pulse pressure	Mortality	30 days	Expected-observed classification
Broderick et al., 1993 [13]	No name	USA (188)	GCS ICH volume	Mortality	30 days	Sensitivity, specificity, PPV
Masé et al., 1995 [14]	No name	Italy (138)	GCS ICH volume IVH	Mortality	30 days	Expected-observed classification
Hemphill et al., 2001 [15]	ICH score	USA (152)	Age GCS ICH volume Infratentorial origin IVH	Mortality	30 days	Descriptive
Cheung et al., 2003 [16]	New ICH score	Hong Kong (142)	IVH NIHSS Pulse pressure Subarachnoid extension Temperature	Mortality Favorable outcome (mRS < 3)	30 days	Sensitivity, specificity, PPV, NPV, Youden index
Godoy et al., 2006 [17]	Modified ICH Scores (mICH-A, −B)	Argentina (153)	Age Comorbidity GCS ICH volume IVH Infratentorial origin	Mortality Favorable outcome (GOS 4–5)	30 days (mort.) 6 months (GOS)	Sensitivity, specificity, PPV, NPV, AUC, Youden index
Weimar et al., 2006 [9]	Essen ICH score	Germany (260)	Age Level of consciousness NIHSS	Functional recovery (BI > 90) Favorable outcome (GOS 4–5, or BI > 50)	100 days (functional recovery) 6 and 12 months (favorable outcome)	Sensitivity, specificity, AUC, external validation (independent cohort, *n* = 173)
Ruiz-Sandoval et al., 2007 [18]	ICH grading scale	Mexico (378)	Age GCS ICH volume (supratentorial or infratentorial) IVH Location	Mortality	In-hospital 30 days	AUC, R^2^
Cho et al., 2008 [19]	Modified ICH score (mICH score)	China (226)	GCS ICH volume IVH or hydrocephalus	Mortality Favorable outcome (GOS 4–5 OR BI > 50)	6 months 12 months (both endpoints at both time points)	AUC, Youden index
Rost et al., 2008 [20]	FUNC score	USA (418)	Age GCS ICH location ICH volume Pre-ICH cognitive impairment	Functional independence (GOS 4–5)	90 days	AUC, External validation (in independent patient cohort from same institution, *n* = 211)
Chuang et al., 2009 [21]	Simplified ICH score	Taiwan (293)	Age Dialysis dependence GCS History of hypertension Serum glucose	Mortality	30 days	Sensitivity, specificity, PPV, NPV, positive/negative likelihood ratios, AUC
Li et al., 2012 [22]	ICH Index (ICHI)	China (227)	Age GCS Glucose WBC	Mortality	In-hospital	AUC
Ji et al., 2013 [23]	ICH functional outcome score (ICH-FOS)	China (1953)	Age GCS Glucose ICH location ICH volume (supratentorial or infratentorial) IVH NIHSS	Mortality Unfavorable outcome (mRS 3–6)	30 days 3, 6, and 12 months	Hosmer-Lemeshow test, AUC, external validation (in independent patient cohort from same institution, *n* = 1302)
Romero et al., 2013 [24]	Spot sign score (SSSc)	USA (131)	CT characteristics Number of spot signs Maximum axial dimension Maximum attenuation	[ICH expansion] Mortality Unfavorable outcome	In-hospital (mortality) 3 months (mortality and unfavorable outcome)	Descriptive
Zis et al., 2015 [25]	Emergency department ICH score (EDICH)	Greece (191)	GCS ICH location ICH volume INR IVH	Mortality	30 days	Sensitivity, specificity, AUC
Gupta et al., 2017 [26]	ICH outomes project (ICHOP) scores (ICHOP_3_, ICHOP_12_)	USA (365)	APACHE II GCS ICH volume NIHSS Pre-morbid mRS	Unfavorable outcome (mRS 4–6)	3 and 12 months	AUC, McFadden R^2^, Cox&Snell R^2^, Nagelkerke R^2^
Sembill et al., 2017 [27]	Max ICH score	Germany (583)	Age IVH Lobar ICH volume NIHSS Non-lobar ICH volume Oral anticoagulation	Unfavorable outcome (mRS 4–6)	12 months	AUC, Youden index
Braksick et al., 2018 [28]	ICH score_FS_	USA (274)	Age FOUR score ICH volume Infratentorial origin IVH	Mortality	30 days	AUC

*Abbreviations*: *AUC* area under the curve (i.e. area under the receiver operating characteristic curve), *ICH* intracerebral hemorrhage, *GCS* Glasgow coma scale, *IVH* intraventricular hemorrhage, *mRS* modified Rankin scale, *BI* Barthel index, *GOS* Glasgow outcome scale, *PPV* positive predictive value, *NPV* negative predictive value, *WBC* white blood count, *NIHSS* National institute of health stroke scale

### Scores and score complexities

All available prognostic scores identified in our literature search contain mortality and/or functional disability as predicted outcome, although the timing of outcome assessment varies substantially (Table 1). More than half of the scoring systems were intended to predict mortality in-hospital or 30 days after the index ICH. In the remainder, the endpoint is functional outcome, invariably dichotomized, at varying times after the ICH, quantified by one or a combination of the following three grading scales: mRS, the Glasgow outcome scale, or the Barthel index (more information available in the online supplement). Figure 1 provides a conceptual illustration of prognostic score complexities for the currently available ICH scores.

### Validation of the Essen-ICH-score

**Fig. 2:**
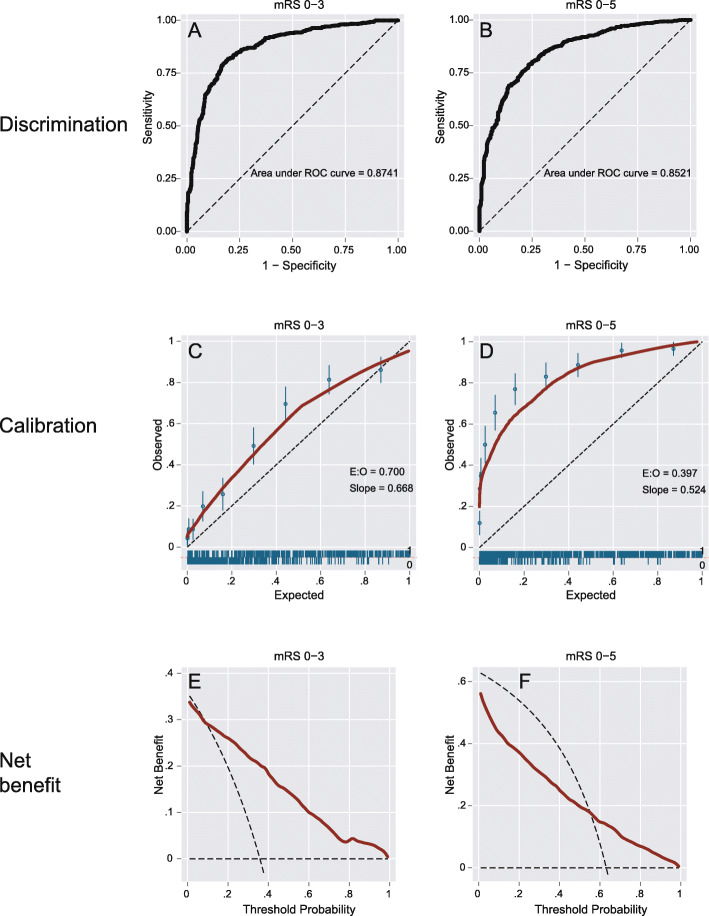
Three validation measures. Discrimination (panel **a** + **b**), calibration (panel **c** + **d**) and net benefit analysis (panel **e** + **f**) are shown for the outcomes mRS score 0–3 (left column), and mRS score 0–5 (=survival, right column). Panels **c** + **d**: the dotted indicates the ideal ratio where expected and observed outcomes are identical. The red line indicates the actually observed ratios. Panels **e** + **f**: the red line indicated the net benefit when using the Essen ICH score on the full range of threshold probabilities, the curved dotted line indicates the net benefit of treating all patients, and the straight dotted line on the x-axis indicated the net benefit of treating no one. Panel **e** shows an overall benefit (mRS 0–3) while panel **f** (mRS 0–5) does not indicate a clear benefit (red line mostly lower than the curved dotted line). Abbreviations: mRS modified Rankin scale, E:O expected/observed ratio, AUC area under the receiver-operating characteristic curve

With the exception of the max-ICH score, which was fully validated recently, none of the scores identified in our literature search provided complete score validation [29]. Here we chose the Essen-ICH-score to illustrate the three methodological pillars of validation discrimination, calibration, and net benefit analysis. We validated the Essen-score in the Erlangen UKER ICH cohort (Fig. 2) [9, 10].

#### Discrimination

The first step of score validation is to understand discriminatory performance, which can be graphically represented by the area under the receiver operator characteristic curve (AUC), and statistically expressed by the concordance statistic (c-statistic). The c-statistic is a measure of the chance that a randomly selected patient with unfavorable outcome had a higher prediction score grade than a patient with favorable outcome. In our example (Fig. 2) the AUC for mRS 0–3 can be considered to be good (0.87, 95% confidence interval, CI, 0.85–0.90). Even though not developed for this endpoint the Essen ICH score’s discriminatory properties regarding survival (mRS 0–5) are good as well (AUC 0.85, CI 0.83–0.87).

#### Calibration

Calibration quantifies the absolute risk of having a certain outcome. Calibration is a necessary addition to discrimination. Absolute risks are illustrated by a calibration curve, where the expected risk (x-axis) needs to be in line with the observed risk (y-axis) for calibration to be perfect. In our example the calibration curve for mRS 0–3 shows that the observed risks of favorable outcomes are greater than what is predicted by the model. The importance of score calibration becomes clear when calibration is plotted for survival as outcome. Because survival is a very broadly defined outcome, it is not surprising that the calibration plots show a strong underestimation of the absolute risks of outcome, despite good discrimination (Fig. 2).

#### Net benefit

The two previous steps have the goal to identify patients who will develop unfavorable outcome and to provide a risk estimate for that. In recent years, a third element has been added that combines discrimination and calibration in a graphical representation, the so-called net clinical benefit curve [30]. There are three main elements of net benefit plots: the net benefit when using the prediction score on the full range of score grades, which is then compared to the net benefit of treating all patients on the one hand (without using a prediction score), and the net benefit of treating no one on the other hand (again without using a prediction score). Our analysis indicates a net benefit from using the Essen ICH score for the prediction endpoint mRS 0–3, but not for the endpoint mRS 0–5 (Fig. 2).

## Discussion

Published in 2018 and including a systematic literature review up to September 2016, a meta-analysis by Gregorio et al. provided a thorough overview on all ICH outcome prediction scores that were available at the time [31]. Interestingly, the authors attempted a direct comparison between machine-learning and conventional regression-based prognostication tools coming to the conclusion that regression-based scores were overall superior. However, scores were mainly judged on their ability to discriminate between dichotomized endpoints (favorable versus unfavorable mRS categories, or alive versus dead), and conceptual problems like the withdrawal of care (WOC) bias or negligence of patient-reported outcomes were not addressed in-depth. In the following discussion our goal was to include those issues that affect prognostic tools at the level of score design and validation.

### Clinical use of currently available scores

The ICH score is probably the most broadly known prognostication tool as it was one of the first available scores and has been extensively studied for different predicted time points [15, 32]. However, there is little data to quantify the actual use of prediction scores in clinical practice. A 2012 survey in 77 German neurointensive care units showed that only 10% of neurologists, 8% of neurosurgeons and none of the surveyed anesthesiologists routinely use the ICH score. Theoretically, the use of the ICH score might be more established in the United States. However, in the same German paper more than 80% of study participants stated to routinely use the GCS and the Hunt&Hess grading scales for subarachnoid hemorrhage. Both scores are frequently used in the United States, indicating that the near neglect of the ICH score among German study participants cannot simply be ascribed to an intercontinental difference in medical culture [5]. Barriers to using clinical scores may include lack of trust in the prognostication instrument or that score calculation may be too time-consuming.

### Problems of current prognostic scores

Prior studies have questioned the clinical utility of ICH prediction scores altogether. In a head-to-head comparison with the GCS scale, the original ICH score and the ICH Grading Scale did not show a net clinical benefit regarding prediction of 30-day mortality [33]. One study in five US American tertiary care centers prospectively compared the accuracy of 3-months outcome predictions between clinicians on the one hand and the ICH and FUNC scores – two of the most frequently used prediction scores – on the other. In this study attending clinician judgment was superior to score predictions [6]. A possible interpretation of these study results is that the human mind integrates more information in its decision-making process than the comparatively simple information contained in the ICH and FUNC scores. For example, both ICH and FUNC score are lacking information on comorbidities, general appearance of the patient, information on code status etc.

#### The withdrawal of care bias

**Fig. 3:**
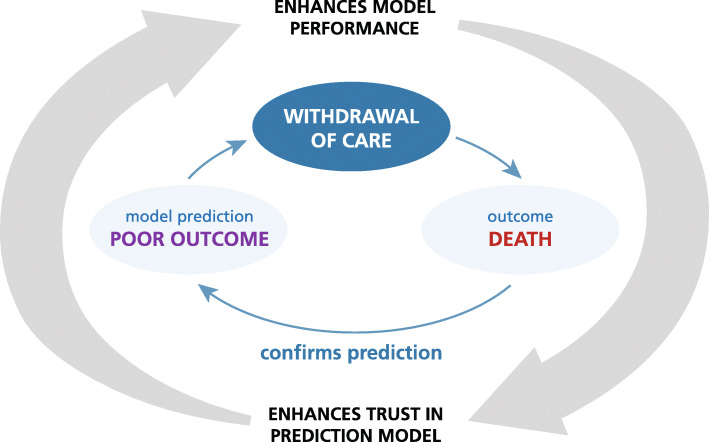
Withdrawal of Care Bias

Another caveat of score-based predictions is the self-fulfilling prophecy bias [7, 33–35]. This bias, also called WOC bias, arises when the decision to withdraw care is based on the prediction that the patient’s outcome will likely be poor. If the WOC decision is based on data provided by a prediction model, the association between predicted and true outcome and thus the performance of the model is artificially strengthened. This in turn may strengthen the model’s influence on clinical decision-making (Fig. 3) [36].

The magnitude of the WOC bias and its effect on a prediction model are difficult to determine. This is because the decision to withdraw care can theoretically go against a model prediction and thus weaken rather than strengthen the performance of the model, for example if care is withdrawn in patients in whom the model predicts a favorable outcome. Furthermore, clinical anticipation of unfavorable outcome may in fact be a correct prediction of the disease course in which case there is no WOC bias [37].

Studies have shown that these considerations are not purely theoretical but that they impact clinical behavior and confound prediction models [38, 39].

The max-ICH score was the first ICH prediction score in which the WOC bias was addressed at the level of score creation. Max-ICH was developed in maximally treated patients excluding those with early care limitations. In the 583 patients included in the study conventional prognostication overestimated the observed mortality by 45% and overestimated the observed unfavorable outcome by 10% [27]. The max-ICH score is the only score that we are aware of that has been fully validated according to the criteria laid out in this review [29].

Whenever prognostic information is being made available to a physician who makes care decisions the WOC bias may continue to exert its influence, even after clinical implementation of a prediction model. Therefore, studying the impact of a prognostic model is difficult. Cluster-randomized controlled trials, comparing treatment outcomes in some centers that use a prediction model with outcomes in other centers that do not use the prediction model, are preferable over classic randomized controlled trials. This is because - by design - cluster randomized trials separate both patients and physicians into the two arms and thus reduce the WOC bias that originates from physicians [40].

The bias originating from the WOC practice is reflected in clinical care guidelines. It is recommended to refrain from early care limitations for at least the first two full days of hospitalization. This recommendation does not apply to patients who have a documented do-not-resuscitate or do-not-intubate or other care limiting orders [41, 42].

#### Simplicity, accuracy, and timing of score assessment

It seems counterintuitive that one prognostic score would meet the goals of both simplicity and prediction accuracy. However, these principles have guided the development of most ICH prediction scores to date. With more advanced technology such as smartphone apps, nowadays even complex prediction models can be easily implemented in clinical practice [43, 44]. However, to date, no smartphone app calculators are available for ICH prediction scores.

The timing of score assessment for most ICH scores is upon admission or briefly after, thereby accommodating the clinical necessity of early prognostication to inform early treatment decisions. Repeat score calculation 5 days into the hospital stay and score calculation using follow up imaging rather than admission imaging has been shown to improve prediction accuracy [45, 46]. In subarachnoid hemorrhage unfavorable long-term outcome despite favorable score prediction, can often be attributed to hospital complications [43]. In ICH, a study in the INTERACT 2 cohort showed that in 17% of patients, the hospital course was complicated by either early or delayed neurologic deterioration, with early worsening being defined as deterioration within 24 h and delayed worsening being defined as deterioration between 24 h and 7 days relative to the index ICH [47]. Imaging correlates of this clinical worsening may be intraparenchymal or intraventricular hemorrhage expansion which, if present, usually occur within 24 h of admission. Both processes have been linked to outcome [48–51]. Development of hydrocephalus and perihemorrhagic edema are examples of dynamic processes that impact outcome at even later time points. Hydrocephalus typically develops days to weeks after the index ICH, while perihemorrhagic edema volume peaks around 2 weeks into the hospital course [52–54]. This lends further support to the idea of repeat prognostication during and towards the end of the hospitalization.

Beyond the acute hospitalization patients with ICH are at increased risk for ischemic stroke as well which may impact long-term outcomes [55, 56]. Current ICH scores do not fully capture the disease and complication burden, which constitutes a potential source of false predictions.

Timing of outcome assessment is not standardized in current prediction scores ranging from in-hospital events to up to 1 year after the index ICH (Table 2). Performance measures cannot be compared between scores if they predict different disease states in time. To clinicians and patient families, three outcome time points seem to be most relevant: the first 7 days (here especially risk of death by the end of the first week), “post-acute” outcome at 90 days (used in most randomized controlled trials), and long-term outcome at 1 year. Early prediction (e.g. within 48 h) is warranted for 7-day mortality risk, while for the 90-days and 1-year time points more input variables across the hospital stay can be gathered. This approach takes advantage of longitudinally updated prognostic information that would otherwise be lost. Similar recommendations have been made for outcome assessment after subarachnoid hemorrhage [57]. Further sources of loss of prognostic information include the common practice of dichotomizing outcome scales (discussed below), and the creation of a prediction score itself. Not only are final score numbers usually the result of rounding, which sacrifices information. They also encode different risk constellations by assigning them identical numbers. For example, on the ICH score an 81-year-old with a 5 ml ICH gets 1 point for his age (> = 80) and no points for his relatively small ICH volume while a 25-year-old with a 35 ml ICH gets 1 point as well (no points for age, 1 point for ICH volume). This assignment of identical scores for likely very different risk constellations can be prevented by the use of continuous scales, where the distance between numbers is equal and proportionally reflects risk.

**Table 2:**
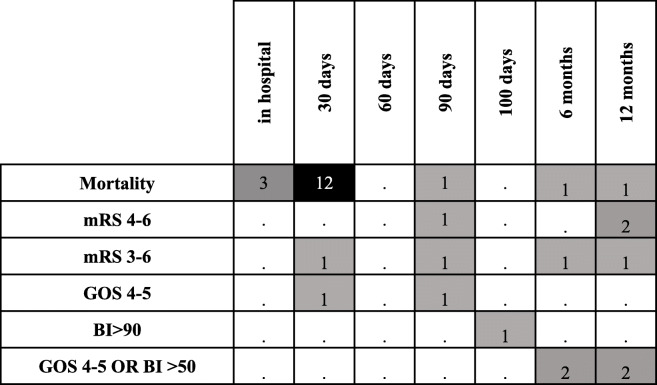
Type and timing of outcome assessment in current ICH prediction scores

Number of scores listed in Table 1 using a given outcome/timing

*mRS* modified Rankin scale, *GOS* Glasgow outcome scale, *BI* Barthel index

### Framework for future ICH prediction scores

A 2019-analysis, conducted jointly by members of the US American and German Neurocritical Care Societies, identified gaps of prognostication in neurocritical care. Gaps in ICH outcomes research include the determination of the best timing of score assessment, addressing the WOC bias as it may confound score derivation, and integration of new predictor variables in future scores [58]. Future scores should thus be derived in maximally treated patient cohorts [27, 59]. Patients with very early care limitations, e.g. patients with DNR/DNI status upon arrival in the emergency room, must be excluded from future score derivation cohorts as aggressive care in these patients would be unethical [59].

### Quality measures for score development and validation

Both the American Heart Association (AHA) and the Neurocritical Care Society (NCS) have emphasized the importance of standardized development and reporting of high-quality prediction models for stroke and neurocritical care [58]. The *Progress Study Group* has outlined quality measures regarding identification of prognostication endpoints, the factors that are associated with these endpoints and creation of prognostic models. Complete validation of prediction models is an indispensable part of score development [40]. The validation of the Essen score described in the results section of this article may serve as an example of a complete score validation, including quantification of discrimination, calibration and net benefit.

### Integration of new predictor variables

Table 3 provides an overview on candidate variables described in the literature for future prediction scores, based on studies linking these variables to outcome after ICH. Of note, these studies may be subject to publication bias [72]. Moreover, an association of a variable with clinical outcome, even if reproducible across multiple studies, does not guarantee that the variable will improve the performance of a prediction model.

**Table 3:** Variables reported in the literature to consider for inclusion into future ICH prediction scores

**Physiologic variables**	APACHE score [26, 60]
	Serum hemoglobin [61]
	Serum neutrophil to lymphocyte ratio [62, 63]
	Cerebral perfusion pressure and partial pressure of oxygen in interstitial brain tissue (PbtO2) [64]
	Serum iron/ferritin/transferrin [65]
	Chronic kidney disease [66]
**Imaging Variables**	IVH expansion [50, 51, 67]
	Peak PHE [68]
	Spot sign/island sign/black hole sign/blend sign [69]
	Non contrast CT-Hypodensities on CT [69, 70]
**EEG variables**	Electrographic seizures [71]
	Periodic discharges [71]

*ICH* intracerebral hemorrhage, *IVH* intraventricular hemorrhage, *PHE* perihemorrhagic edema, *CT* computed toography

### Beyond binary outcomes: modified Rankin scale and patient-reported outcomes

The mRS is the most frequently used outcome measure in stroke trials. It has been criticized for being poorly reproducible, for having score grades that are not in proportion with each other, for not distributing patients normally among its score values, and for being heavily focused on mobility rather than cognitive or social functioning [73, 74]. Furthermore, dichotomization – which is also common practice in current ICH prediction scores – leads to loss of prognostic information (Table 2). Its use has been fueled by statistical packages which often contain logistic regression for binary outcomes. Both statistically and clinically, dichotomization is an unhelpful over-simplification.

Toward the goal of patient-centered outcomes quantification, PROMs have been proposed. The patient reported outcomes information system (PROMIS) is one example of a detailed assessment tool, designed for multiple disease entities. It includes domains such as physical function, social roles, pain, fatigue, anxiety and sleep [75]. A similar tool, specifically for the use in neurological disorders, is the Quality of Life in Neurological disorders (NeuroQuol) tool set, consisting of brief PROM surveys across 13 domains [76]. Further frequently used PROM scales include the EQ-5D, the Sickness Impact Profile, the Telephone Interview for Cognitive Status (TICS) and the SF-36. The prospective Patient Reported Outcomes in Stroke Care (EPOS, NCT03795948) study as well as the coordinated treatment of stroke patients with patient-orientated outcome measurement (StroCare, NCT04159324) study will hopefully further characterize the role PROMs in stroke-related outcomes research [77, 78]

### Pragmatic prognostication in clinical practice

The 2015 AHA guidelines on the management of ICH recommend the use of a severity score at the time of presentation, such as the NIHSS or ICH score. However, basing the prognosis on a single scoring system is discouraged [41]. The NCS, and the American Academy of Neurology affirm the AHA guideline, but neither these two societies nor the Society of Critical Care Medicine provide their own dedicated ICH guidelines.

Despite the above-mentioned shortcomings of available ICH outcome prediction scores, it may be reasonable to approach the prognosis using one of the current scores, ideally one that underwent the most thorough testing over the years, i.e. the ICH score or the Essen score. As a principle, it is recommended to never base the prognosis on one score result alone [41] but rather to consider all information relevant to the patient’s best interest (Table 4).

**Table 4:** Proposal of a clinical approach to handling prognostic information in patients with severe ICH and “full code” status

**First approach to the patient**	Clarify code status. Be aware of your own biases. Especially in patients with large ICH volumes and/or IVH extension and/or hydrocephalus do not reflexively, consciously or subconsciously, provide sub-maximal care. Unless patients are at immediate risk of dying or fulfill criteria for brain death, provide maximal therapy, at least until contact with family is established and/or direct access to patient’s living will.
**First family communication**	Establish a relationship of trust and try to speak with close family members in person rather than on the phone if possible. Inquire whether a documented living will exists. Provide objective information. Avoid choice of words or implicit communication elements that suggest a likely clinical outcome (it is reasonable however to say that the disease is “severe” or “potentially life-threatening” if that is the case). Assess the family’s overall understanding of the situation, explain the disease, leave room for questions.
**Score prognostication**	Calculate the likelihood of unfavorable outcome using a recently validated prediction score. Be aware of the shortcomings of current prognostication tools, especially the lack of incorporation of worsening or improvement of the patient over time. Never make a definitive recommendation/decision solely based on the score results.
**Second family communication**	Use the score information to give the family a sense how patients with a similar disease severity have done in the past. Avoid confronting patients/families with numbers. In rare cases, if the educational level of family allows it, explain biases and shortcomings of our current prediction tools. Explain that aggressive therapy may make a relative outcome difference even in situations where moderate to severe disability is very likely.

### Limitations

This review has limitations. First, the reader should be aware that, using only pubmed as a data source and conducting the literature search over a limited time period, our review can be considered a *rapid review* but does not fulfill all criteria required for a *systematic review*. Second, scores developed in other disease entities and then used for ICH prediction are not included in our paper. This is also true for clinical scales such as the GCS or the NIHSS which have been used in some publications to predict outcome after ICH. These publications are partially referenced here but are not included in the literature review portion. Third, when demonstrating score validation using the Essen ICH score, the endpoint (Barthel Index) that the Essen score was developed for was not available in the validation cohort. Strictly speaking, we did not validate the original Essen score but a variation of the Essen score using the mRS instead of the Barthel index as outcome. Therefore further validation of the original score would be needed if the Essen score were to be used for clinical decision making.

## Conclusion

Despite prediction score fatigue among clinicians, prognostication in patients with ICH remains crucially important. It may improve clinical care by providing information on the most probable outcome, which can then be aligned with what is medically feasible and what the patient wants. Prediction models may also be used to risk-stratify participants of clinical trials, which may ultimately facilitate implementation of new therapies. Currently available prediction scores do not have a unified modeling approach or type or timing of outcome assessment. In this article we describe necessary premises for the development of more reliable ICH prediction scores. Future scores need to be developed in maximally treated patient cohorts to ameliorate the WOC bias. The timing of score and outcome assessments needs to be standardized: we propose capturing predictors within 24 h after the index bleed for in-hospital prognostication, and re-capturing information at 7 days to determine prognosis at 90 days. We propose to consistently use the mRS as a disability scale, and to add PROMs to obtain a complete picture of outcomes that matter to patients and families. Lastly, score development requires rigorous validation including discrimination, calibration and net benefit analyses. Until improved prognostic scores are available, we encourage clinicians to learn about the shortcomings of current ICH scores and use them with these caveats in mind.

## Supplementary Information

**Additional file 1.**

## Data Availability

The dataset analyzed during the current study is not publicly available. Upon reasonable request questions regarding the current analysis will be answered in detail by the corresponding author.
