# Supplementary material for: Prognostication after intracerebral hemorrhage: a review
Source: Neurol Res Pract. 2021 May 3;3:22. doi: 10.1186/s42466-021-00120-5 (PMC8091769; doi:10.1186/s42466-021-00120-5)
Supplement: Supplementary file 1 — Additional file 1. [file 42466_2021_120_MOESM1_ESM.docx]

**Supplementary Data**

Witsch J, Siegerink B, Nolte C, Sprügel M, Steiner S, Endres M, Huttner H:

Prognostication after intracerebral hemorrhage: a review

**eMethods.**

Pubmed Search Algorithm

Inclusion and Exclusion Criteria

**eFigure 1.** Literature Search Flow Diagram

**eResults.**

**eReferences.**

**eMethods.**

The search algorithm was designed with the support of a medical librarian and adjusted so that it would detect nine sentinel papers contained in a prior review on the topic.(Chu & Hwang, 2016)

We searched Pubmed up to December 31, 2020, with the terms:

*((("Intracerebral Hemorrhage"[Mesh] or "intracerebral hemorrhage" or “intraparenchymal hemorrhage” or “intraventricular hemorrhage”))) AND ((((((predict* or outcome* or prognos*)) AND (score* or scale* or model*))) OR risk stratification))*

**Inclusion and exclusion criteria**

Inclusion criteria were: clinical studies on spontaneous intracerebral hemorrhage in patients 18 years or older. We excluded studies on ICH secondary to vascular malformation, traumatic ICH, primary other intracranial hemorrhage with ICH component, as well as studies on hemorrhage secondary to thrombolysis for ischemic stroke. Studies on mixed patient cohorts (e.g. traumatic and spontaneous ICH) were excluded. Case reports and case series on less than 10 patients were excluded as well.

All studies potentially fulfilling the inclusion criteria based on review of their title and abstract were read in full length. Among these studies more specific exclusion criteria were the following:

**Specific reasons for exclusion**

**Relevance**: Study aim not relevant. **(R)**

**Design**: Not reporting on a prognostication score or not a primary research article. **(D)**

**Population**: Study conducted in an ineligible/mixed population **(P)**

**Intervention**: The primary aim of the study was to prognosticate outcome after an intervention, e.g. surgical hematoma removal. **(I)**

**Outcomes**: No relevant outcomes reported **(O)**

**Language**: Paper not in English. **(L)**

**Studies excluded after reading the full paper / reason:**

- Anderson et al., 1994(Anderson, Jamrozik, Broadhurst, & Stewart-Wynne, 1994) / mixed cohort (ischemic and hemorrhagic stroke) **(P)**
- Edwards et al., 1995(Edwards, Chen, & Diringer, 1995) / mixed cohort (ischemic and hemorrhagic stroke) **(P)**
- Ang et al., 2008(Ang, Chan, Lee, & Ng, 2008) / development of several models for mortality prediction but no clinic score development **(D)**
- Di Napoli et al., 2011(Di Napoli et al., 2011) / addition to inflammatory biomarkers with ICH score components in one model, but no new score developed **(D)**
- Rathor et al., 2012(Rathor et al., 2012) / No score developed **(D)**
- Zahuranec et al., 2012(Zahuranec et al., 2012) / No score developed **(D)**
- Smith et al., 2013(Smith et al., 2013) / mixed cohort (ischemic and hemorrhagic stroke) **(P)**
- Tao et al., 2014(Tao, Wang, Schlaug, Liu, & Selim, 2014) / addition of imaging characteristic to ICH score, no new score developed **(D)**
- Ziai et al., 2015(Ziai et al., 2015) / selected cohort: development of survival score for subgroup of ICH patients (severely sick) **(P)**
- Tan et al., 2016(Tan et al., 2016) / model development without clinical score **(D)**
- Fallenius et al., 2017(Fallenius, Skrifvars, Reinikainen, Bendel, & Raj, 2017) / developed two reference models to compare with physiological ICU scores (APACHE, SAPS, SOFA). No score developed. **(D)**
- Ding et al., 2018(Ding et al., 2018) / unclear cohort inclusion criteria, likely selected subgroup (patient with hypertensive ICH only) **(D)**
- He et al., 2020(He et al., 2020). No score developed. **(D)**
- Kimura et al., 2020(Kimura et al., 2020). No score developed. **(D)**

**eFigure 1.** Literature Search Flow Diagram


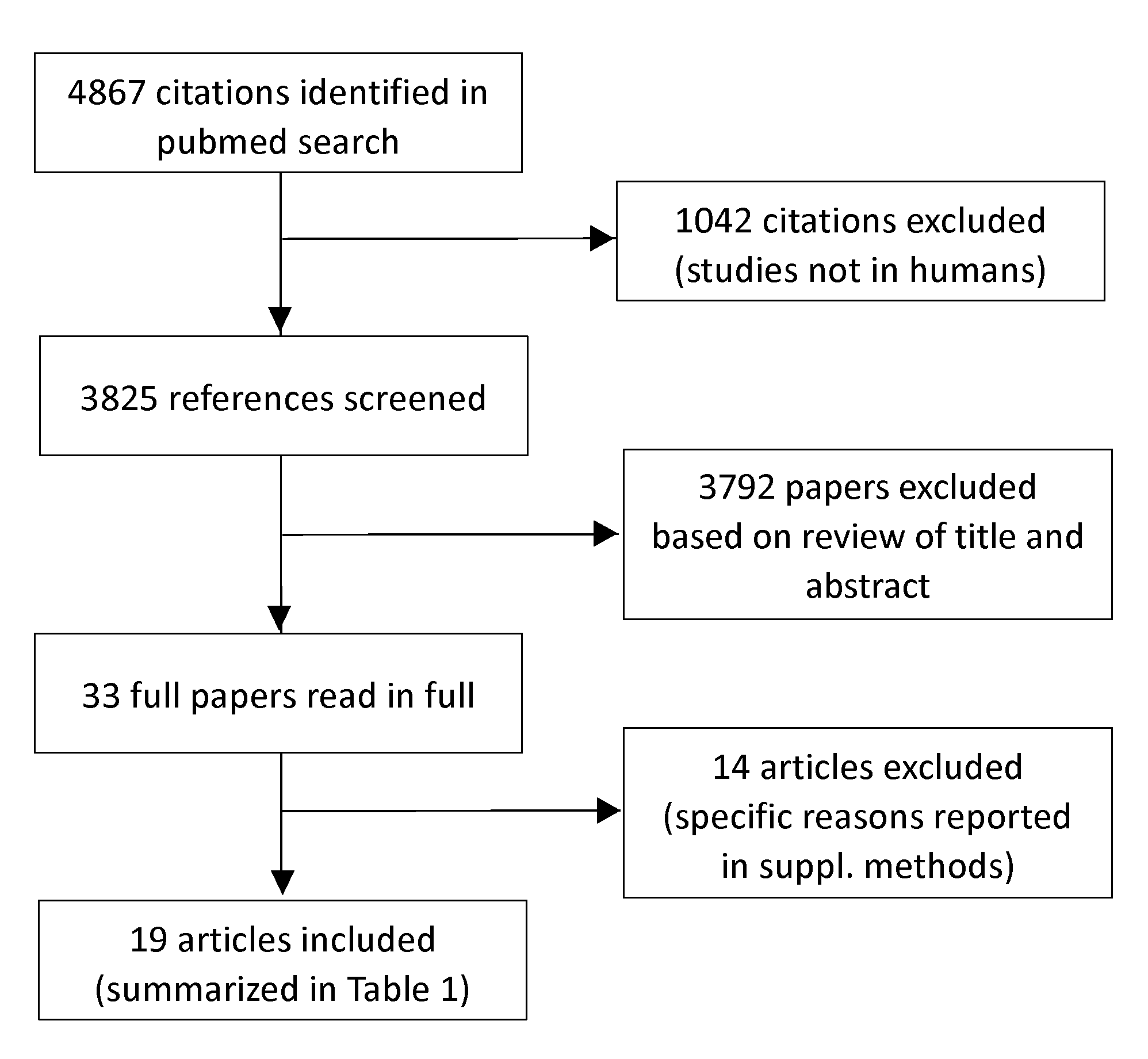


**eResults**

Prognostication of disability

The median number of score components is 5, with a range between 2 and 7 predictor variables. 13/19 scores include level of consciousness and ICH volume as score components, 9/19 use level of consciousness, ICH volume and IVH as score components, and 6/19 include LOC, ICH volume, IVH and age. In 7 scoring systems a physiological variable was used(Cheung & Zou, 2003; Chuang, Chen, Peng, & Peng, 2009; Gupta et al., 2017; Li et al., 2012; Tuhrim et al., 1988; Tuhrim et al., 1991; Zis et al., 2014) (pulse pressure, serum glucose WBC, or INR) with on score integrating the APACHE II physiological score as one score variable. The majority of scoring systems are derived from multivariate regression analyses identifying factors associated with functional outcome.

**eReferences.**

Anderson, C. S., Jamrozik, K. D., Broadhurst, R. J., & Stewart-Wynne, E. G. (1994). Predicting survival for 1 year among different subtypes of stroke. Results from the Perth Community Stroke Study. *Stroke, 25*(10), 1935-1944. doi:10.1161/01.str.25.10.1935

Ang, B. T., Chan, S. P., Lee, K. K., & Ng, I. (2008). Prediction of early mortality in primary intracerebral hemorrhage in an Asian population. *Acta Neurochir Suppl, 102*, 299-303. doi:10.1007/978-3-211-85578-2_56

Cheung, R. T., & Zou, L. Y. (2003). Use of the original, modified, or new intracerebral hemorrhage score to predict mortality and morbidity after intracerebral hemorrhage. *Stroke, 34*(7), 1717-1722. doi:10.1161/01.Str.0000078657.22835.B9

Chu, S. Y., & Hwang, D. Y. (2016). Predicting Outcome for Intracerebral Hemorrhage Patients: Current Tools and Their Limitations. *Semin Neurol, 36*(3), 254-260. doi:10.1055/s-0036-1581992

Chuang, Y. C., Chen, Y. M., Peng, S. K., & Peng, S. Y. (2009). Risk stratification for predicting 30-day mortality of intracerebral hemorrhage. *Int J Qual Health Care, 21*(6), 441-447. doi:10.1093/intqhc/mzp041

Di Napoli, M., Godoy, D. A., Campi, V., del Valle, M., Pinero, G., Mirofsky, M., . . . Rabinstein, A. A. (2011). C-reactive protein level measurement improves mortality prediction when added to the spontaneous intracerebral hemorrhage score. *Stroke, 42*(5), 1230-1236. doi:10.1161/strokeaha.110.604983

Ding, W., Gu, Z., Song, D., Liu, J., Zheng, G., & Tu, C. (2018). Development and validation of the hypertensive intracerebral hemorrhage prognosis models. *Medicine (Baltimore), 97*(39), e12446. doi:10.1097/md.0000000000012446

Edwards, D. F., Chen, Y. W., & Diringer, M. N. (1995). Unified Neurological Stroke Scale is valid in ischemic and hemorrhagic stroke. *Stroke, 26*(10), 1852-1858. doi:10.1161/01.str.26.10.1852

Fallenius, M., Skrifvars, M. B., Reinikainen, M., Bendel, S., & Raj, R. (2017). Common intensive care scoring systems do not outperform age and glasgow coma scale score in predicting mid-term mortality in patients with spontaneous intracerebral hemorrhage treated in the intensive care unit. *Scand J Trauma Resusc Emerg Med, 25*(1), 102. doi:10.1186/s13049-017-0448-z

Gupta, V. P., Garton, A. L. A., Sisti, J. A., Christophe, B. R., Lord, A. S., Lewis, A. K., . . . Connolly, E. S., Jr. (2017). Prognosticating Functional Outcome After Intracerebral Hemorrhage: The ICHOP Score. *World Neurosurg, 101*, 577-583. doi:10.1016/j.wneu.2017.02.082

He, X. W., Chen, M. D., Du, C. N., Zhao, K., Yang, M. F., & Ma, Q. F. (2020). A novel model for predicting the outcome of intracerebral hemorrhage: Based on 1186 Patients. *J Stroke Cerebrovasc Dis, 29*(8), 104867. doi:10.1016/j.jstrokecerebrovasdis.2020.104867

Kimura, Y., Miwa, K., Takasugi, J., Oyama, N., Todo, K., Sakaguchi, M., . . . Sasaki, T. (2020). Total small vessel disease score and functional outcomes following acute intracerebral hemorrhage. *J Stroke Cerebrovasc Dis, 29*(8), 105001. doi:10.1016/j.jstrokecerebrovasdis.2020.105001

Li, Y. F., Luo, J., Li, Q., Jing, Y. J., Wang, R. Y., & Li, R. S. (2012). A new simple model for prediction of hospital mortality in patients with intracerebral hemorrhage. *CNS Neurosci Ther, 18*(6), 482-486. doi:10.1111/j.1755-5949.2012.00320.x

Rathor, M. Y., Rani, M. F., Jamalludin, A. R., Amran, M., Shahrin, T. C., & Shah, A. (2012). Prediction of functional outcome in patients with primary intracerebral hemorrhage by clinical-computed tomographic correlations. *J Res Med Sci, 17*(11), 1056-1062.

Smith, E. E., Shobha, N., Dai, D., Olson, D. M., Reeves, M. J., Saver, J. L., . . . Schwamm, L. H. (2013). A risk score for in-hospital death in patients admitted with ischemic or hemorrhagic stroke. *J Am Heart Assoc, 2*(1), e005207. doi:10.1161/jaha.112.005207

Tan, G., Hao, Z., Lei, C., Chen, Y., Yuan, R., Xu, M., & Liu, M. (2016). Subclinical change of liver function could also provide a clue on prognosis for patients with spontaneous intracerebral hemorrhage. *Neurol Sci, 37*(10), 1693-1700. doi:10.1007/s10072-016-2656-0

Tao, W. D., Wang, J., Schlaug, G., Liu, M., & Selim, M. H. (2014). A comparative study of fractional anisotropy measures and ICH score in predicting functional outcomes after intracerebral hemorrhage. *Neurocrit Care, 21*(3), 417-425. doi:10.1007/s12028-014-9999-2

Tuhrim, S., Dambrosia, J. M., Price, T. R., Mohr, J. P., Wolf, P. A., Heyman, A., & Kase, C. S. (1988). Prediction of intracerebral hemorrhage survival. *Ann Neurol, 24*(2), 258-263. doi:10.1002/ana.410240213

Tuhrim, S., Dambrosia, J. M., Price, T. R., Mohr, J. P., Wolf, P. A., Hier, D. B., & Kase, C. S. (1991). Intracerebral hemorrhage: external validation and extension of a model for prediction of 30-day survival. *Ann Neurol, 29*(6), 658-663. doi:10.1002/ana.410290614

Zahuranec, D. B., Sanchez, B. N., Brown, D. L., Wing, J. J., Smith, M. A., Garcia, N. M., . . . Lisabeth, L. D. (2012). Computed tomography findings for intracerebral hemorrhage have little incremental impact on post-stroke mortality prediction model performance. *Cerebrovasc Dis, 34*(1), 86-92. doi:10.1159/000339684

Ziai, W. C., Siddiqui, A. A., Ullman, N., Herrick, D. B., Yenokyan, G., McBee, N., . . . Hanley, D. F. (2015). Early Therapy Intensity Level (TIL) Predicts Mortality in Spontaneous Intracerebral Hemorrhage. *Neurocrit Care, 23*(2), 188-197. doi:10.1007/s12028-015-0150-9

Zis, P., Leivadeas, P., Michas, D., Kravaritis, D., Angelidakis, P., & Tavernarakis, A. (2014). Predicting 30-day case fatality of primary inoperable intracerebral hemorrhage based on findings at the emergency department. *J Stroke Cerebrovasc Dis, 23*(7), 1928-1933. doi:10.1016/j.jstrokecerebrovasdis.2014.02.006
